# Complete mitochondrial genome of the *Salvelinus malma* sp. (Salmoniformes, Salmonidae) with phylogenetic consideration

**DOI:** 10.1080/23802359.2017.1403865

**Published:** 2017-11-28

**Authors:** Liguo Yang, Fanxing Meng, Rixin Wang, Ge Shi

**Affiliations:** a College of Marine Science, Zhejiang Ocean University, Zhoushan, China;; b School of Marine Sciences, Ningbo University, Ningbo, China

**Keywords:** *Salvelinus malma* sp.;· mitochondrion;· genome

## Abstract

The complete mitochondrial genome sequence of Chinese native Dolly Varden (*Salvelinus malma* sp.) was sequenced. It was 16,652-nucleotide in length and consisted of 13 protein-coding genes, 2 ribosomal RNA genes, 22 transfer RNA genes, and 2 non-coding regions (L-strand replication origin and control region), showing conserved gene arrangement with most vertebrates. The phylogenetic analysis based on *Cytb* genes of Chinese native as well as four other Dolly Varden subspecies showed that Chinese native *S. malma* sp. had close relationships with *S. curilus* (formerly named as *S. malma krascheninnikova*) and *S. malma miyabe*. This result did not support the monophyletic relationship of Dolly Varden chars. Further studies are needed to determine the phylogenetic position of Chinese native Dolly Varden.

Dolly Varden is a large anadromous or freshwater char (*Salvelinus malma*). It inhabits in the streams of north-western North America, Russian Far East, Korea, Japan, as well as in Northeast of China (Armstrong and Morrow 1980). However, little genetic information of Chinese native Dolly Varden (*Salvelinus malma* sp.) is available. In this study, we sequenced and described the complete mitochondrial genome of *S. malma* sp. native in China for the first time.

The specimen was collected from the upper Yalu River in Jilin Province, China. The samples were stored in −80 °C in Research Institute of Marine Biotechnology and life Health, Ningbo University, Ningbo. Total DNA was extracted from dorsal fin following TIANamp Marine Animals DNA Kit (Tiangen, China). Totally 28 pairs of primers were used, in which 16 pairs of primers were retrieved from *Gonostoma gracile* (Miya and Nishida 1999) and the remaining primers were designed based on public mitochondrial genome sequences of *Salvelinus albus* (white char), *S. fontinalis* and *S. curilus* (with accession number of NC_028018, NC_000860, NC_024585, respectively).

The sequenced fragments were *de novo* assembled into complete mitogenome and annotated by comparing with published genome sequences of other vertebrate species using Mega5.1 (Tamura et al. 2011) and MitoFish (Iwasaki et al. 2013). Finally, a physical map of *S. malma* sp. mitogenome was generated and uploaded to GenBank with accession number MF680544.

The complete mitogenome of Chinese native Dolly Varden was 16,652 bp in length. The genomic organization was identical to those of typical vertebrate mitochondrial genomes, including two rRNA genes, 13 protein-coding genes, 22 tRNA genes, a light-strand replication origin (OL), and a putative control region (CR). The overall base composition was 28.1% of A, 26.4% of T, 28.5% of C, and 17.0% of G with a slight A + T bias (54.5%) like other vertebrate mitochondrial genomes. The 13 protein-coding genes totally encoded 3808 amino acids and their base composition was 25.7%, 9.0%, 29.0%, and 16.3% for A, T, C, and G, respectively. The features mentioned above were accordant with typical *Salvelinus* fish mitogenome (Salmenkova et al. 2009; Balakirev et al. 2016).

For the 13 protein-coding genes, 12 genes started with ATG while only *COI* started with GTG. Six genes shared the termination codon TAA (*COI*, *ATPase8*, *ND1*, *ND2*, *ND5*, and *ND4L*), one with TAG (*ND6*), the remaining with incomplete stop codon (*COII*, *COIII*, *ND3*, *ND4*, *ATPase6*, and *Cytb*). This feature was common among vertebrate mitochondrial protein-coding genes (Cheng et al. 2010). Chinese native *S. malma* sp. had two non-coding regions, the L-strand replication origin region (36 bp) located between tRNA-Asn and tRNA-Cys, and the control region located within the tRNA-Pro and tRNA-Phe. The 13 protein-encoding genes of Chinese native *S. malma* sp. mitochondrion encoded 3808 amino acids with stop codons included. Leucine was the most frequent amino acid while cystine was the least frequent. CTT was the most frequent used codon (4.46%) and AAG was the least (0.11%), showing similar features with those of other vertebrates (Miya et al. 2003).

To explore the phylogenetic position of Chinese native Dolly Varden, phylogenies based on maximum likelihood (ML) and Bayesian method were constructed using public *Cytb* genes involving other nine Salmonidae species. Two species, *Oncorhynchus clarkii henshawi* and *O. mykiss* × *Salmo salar*, were used as outgroups. The nucleotide sequences were aligned by ClustalX with default settings and calculated the best-fit nucleotide model (TrN + I) (Tamura and Nei 1993) by jModelTest 0.1 (Posada 2008). Then the ML and Bayesian analyses were conducted by PhyML3.1 (Guindon et al. 2010) and MrBayes (Huelsenbeck and Ronquist 2001) and validated by 1000 bootstraps and posterior probability, respectively. The ML and Bayesian methods resulted in the same phylogeny, showing that Chinese native Dolly Varden (*S. malma* sp.) had a close relationship with *S. curilus* (a.k.a. *S. malma krascheninnikova*) and *S. malma miyabe* (Figure 1), forming an Asian group while *S. malma lordi* and *S. malma malma*, together with *S. albus* and *S. kuznetzovi* forming another group. It did not support the monophyletic relationship of *S. malma* fish. Further investigations on morphological and molecular comparisons are needed to resolve the phylogenetic position of Chinese native Dolly Varden.

**Figure 1.**
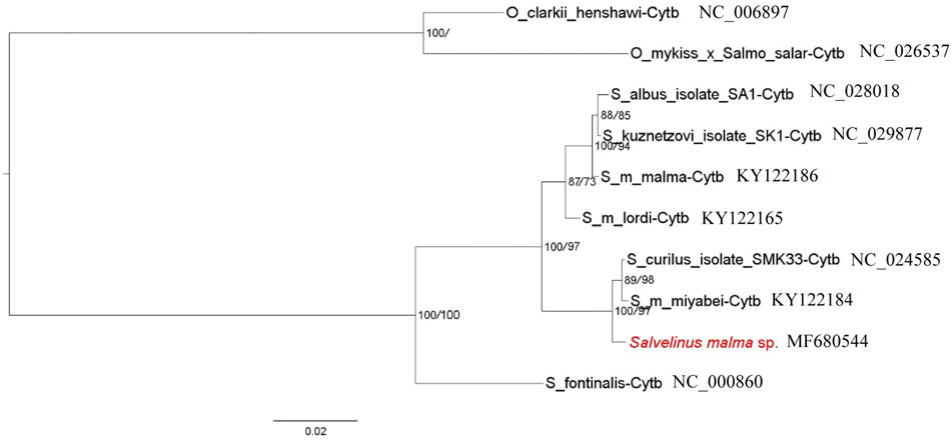
Phylogenetic tree of Dolly Varden chars as inferred from the nucleotide sequences of partial *Cytb* genes. The phylogeny was reconstructed by maximum likelihood and Bayesian method, respectively. The percent values of bootstrap/posterior probability were showed at each node. And the Chinese Dolly Varden was shown in red.
